# Psychological distress in relation to site specific cancer mortality: pooling of unpublished data from 16 prospective cohort studies

**DOI:** 10.1136/bmj.j108

**Published:** 2017-01-25

**Authors:** G David Batty, Tom C Russ, Emmanuel Stamatakis, Mika Kivimäki

**Affiliations:** 1 Department of Epidemiology and Public Health, University College, London, UK; 2 Centre for Cognitive Ageing and Cognitive Epidemiology, University of Edinburgh, Edinburgh, UK; 3 Division of Psychiatry, Centre for Clinical Brain Sciences, University of Edinburgh, Edinburgh, UK; 4 Charles Perkins Centre, Faculty of Health Sciences, University of Sydney, Sydney, Australia

## Abstract

**Objective** To examine the role of psychological distress (anxiety and depression) as a potential predictor of site specific cancer mortality.

**Design** Pooling of individual participant data from 16 prospective cohort studies initiated 1994-2008.

**Setting** Nationally representative samples drawn from the health survey for England (13 studies) and the Scottish health survey (three studies).

**Participants** 163 363 men and women aged 16 or older at study induction, who were initially free of a cancer diagnosis, provided self reported psychological distress scores (based on the general health questionnaire, GHQ-12) and consented to health record linkage.

**Main outcome measure** Vital status records used to ascertain death from 16 site specific malignancies; the three Scottish studies also had information on cancer registration (incidence).

**Results** The studies collectively contributed an average of 9.5 years of mortality surveillance during which there were 16 267 deaths (4353 from cancer). After adjustment for age, sex, education, socioeconomic status, body mass index (BMI), and smoking and alcohol intake, and with reverse causality (by left censoring) and missing data (by imputation) taken into account, relative to people in the least distressed group (GHQ-12 score 0-6), death rates in the most distressed group (score 7-12) were consistently raised for cancer of all sites combined (multivariable adjusted hazard ratio 1.32, 95% confidence interval 1.18 to 1.48) and cancers not related to smoking (1.45, 1.23 to 1.71), as well as carcinoma of the colorectum (1.84, 1.21 to 2.78), prostate (2.42, 1.29 to 4.54), pancreas (2.76, 1.47 to 5.19), oesophagus (2.59, 1.34 to 5.00), and for leukaemia (3.86, 1.42 to 10.5). Stepwise associations across the full range of distress scores were observed for colorectal and prostate cancer.

**Conclusion** This study contributes to the growing evidence that psychological distress might have some predictive capacity for selected cancer presentations, in addition to other somatic diseases.

## Introduction

Although the notion of a link between mental health and physical health was first advanced centuries ago,^1^ the discovery of pathogenic causes for many diseases led to an extended period of quiescence in this field. In recent decades, most research has been conducted in the context of cardiovascular disease, with growing evidence implicating the psychological factors of psychosocial stress,^2^ cognitive function,^3^ and selected personality types (particularly neuroticism and conscientiousness)^4^ as potentially having roles at various stages of the disease process, including acting as predictive factors, markers of undiagnosed pathology, triggers of clinical events in individuals with subclinical disease, or a consequence of diagnosed somatic disease.^5^

The predictive capacity of a further psychological factor—psychological distress (symptoms of depression and anxiety)—in the development of cardiovascular disease has also been explored, with meta-analyses showing positive relations with risk of coronary heart disease^6^ and stroke.^7^ ^8^ Like cardiovascular disease, cancer is a major cause of death and morbidity,^9^ yet few studies have examined its links with distress. Various mechanisms have been implicated in linking psychological distress with cancer. Recurrent exposure to emotional distress could diminish natural killer cell function, which has been implicated in tumour cell control.^10^ Of particular relevance to hormone related cancers is the suggestion that symptoms of depression could lead to dysregulation of the hypothalamic pituitary adrenal (HPA) axis, increase cortisol concentrations and immunological and inflammatory responses, and inhibit DNA repair, so unfavourably impacting on multiple cancer defence processes.^11^ With there also being evidence that, relative to their non-distressed counterparts, people with distress symptoms are more likely to smoke, be sedentary, have an unfavourable diet, and become obese, distress could also increase the likelihood of cancer indirectly through these lifestyle related risk factors.^12^

The few existing prospective cohort studies, which provide the best test of an association in observational epidemiology, are generally small in size and show highly discordant findings with positive, null, and even inverse associations between distress and cancer reported.^13^ Other major gaps in understanding include the extent to which associations might be dependent on site—as cancer is not a single disease entity—and whether any apparent gradient could be generated by reverse causality—that is, distress might be a consequence of the early stages of the malignancy rather than a potential predictor. It is also the case that some studies are insufficiently well characterised to explore alternative explanations for the observed associations, including confounding by health behaviours, socioeconomic status, and systemic inflammation.

In view of the limitations of the existing evidence base, we pooled unpublished individual participant data from 16 community based prospective cohort studies that used the same methods to ascertain psychological distress, covariates, and cancer. In contrast to the more common study level meta-analytical technique,^13^ the use of unpublished raw data across a series of studies provides more precise estimates of the associations between uniformly defined risk markers and disease; a consistent approach to statistical control for plausible covariates and subgroup analyses; and a method that is less likely to suffer from publication bias, which besets modern epidemiology. While individual participant meta-analysis has been extensively applied to the study of the role of physiological factors for risk of disease,^14^ ^15^ to the best of our knowledge, this is the first pooling of individual participant data on psychological distress, as opposed to major depression,^13^ and the risk of specific malignancies. In view of some of the described mechanisms potentially linking distress with selected malignancies, we hypothesised positive associations between distress and cancer for hormone related (breast, prostate, ovary) and lifestyle related (lung, colorectal, pancreas, oesophagus, stomach) cancers.

## Methods

Participants were taken from the health survey for England (HSE)^16^ and Scottish health surveys(SHS),^17^ a series of geographically representative health examinations of people from the general population. Between 1994 and 2008, 16 independent, cross sectional, and methodologically near identical studies were conducted on either an annual (HSE; n=13) or occasional basis (SHS; n=3). The original purpose of these studies was to monitor secular trends in health and related behaviours. A total of 199 504 men and women, aged 16-107 at baseline, were surveyed, with consenting study members linked to national health registers for vital status and, in the case of the SHS only, incidence of cancer.

### Measurement of psychological distress

During a household visit, interviewers administered computer assisted personal interviewing modules that included the 12 item version of the general health questionnaire (GHQ-12).^18^ A widely used measure of psychological distress in population studies, the GHQ-12 comprises items capturing symptoms of depression and anxiety over the previous four weeks. Item response is based on a 4 point scale that signals the presence of a symptom (“not at all”/“same as usual” were give a score of 0; “more than usual”/“much more than usual,” a score of 1). Consistent with our previous analyses,^8^ ^19^ ^20^ ^21^ we divided people into four groups: asymptomatic (score 0), subclinically symptomatic (1-3), symptomatic (4-6), and highly symptomatic (7-12). The GHQ-12 has been validated against standardised psychiatric interviews.^22^ ^23^

### Measurement of cancer at baseline and covariate data

Cancer at baseline was based on self report (HSE), or self report and cancer registration (SHS). The validity of self reported cancer data has been validated against standard records from cancer registries. Although there is evidence that increased age and lower socioeconomic status are associated with lower levels of agreement,^24^ the ability of people to report a past diagnosis of cancer accurately seems to be sufficiently high for specific cancer sites of relevance to our study, such as breast, prostate, lung, and colon.^25^ Height and weight were measured directly, and body mass index (BMI) computed. A BMI of ≥30 was used to denote obesity.^26^ The following characteristics were self reported: age on leaving full time education (minimum allowable age for leaving secondary school was 12-16 depending on epoch), smoking status (not a current smoker; or <5, 5-10, 10-15, 15-20, and >20 cigarettes/day), frequency of alcohol consumption (never drinker, ex-drinker, 1-2 drinks a month, 1-4 drinks a week, or ≥5 drinks a week),^16^ ^17^ and physical activity (five or more occasions of moderate to vigorous physical activity a week).

Other covariates were collected only in certain survey years. Area-based socioeconomic deprivation was derived by linking study member postcode with the index of multiple deprivation (HSE: 2001-6; SHS: 1995, 1998, 2003); serum C reactive protein, a marker of systemic inflammation, was measured from blood samples drawn by a nurse at a second home visit^27^ (HSE: 1998, 2003-6; SHS: 1998, 2003); physical activity was self reported (HSE: 1994, 1997-99, 2003, 2004, 2006, 2008; SHS: 1995, 1998, 2003)^28^; and from data on quantity of weekly alcohol intake in surveys (HSE: 1994-95, 1997-98, 1999-2000, 2001; SHS: 2003)^29^ we categorised study members into harmful drinkers (>14 units/week^30^).

### Outcome ascertainment: cancer mortality and incidence

Study members were linked to the National Health Service (NHS) central registries at Southport and Dumfries, UK, the procedures of which provide the vital status of study members and, when applicable, causes of death, which included cancer. Cancers deaths were denoted by cancer recorded as the underlying cause of death on the death certificate (as opposed to contributing cause). Cancer registrations for a diagnosis of a non-fatal malignancy (incidence) were also available for the three Scottish studies through the Scottish cancer registry. All cancers combined were denoted by ICD-9 (international classification of disease, ninth edition)^31^ codes 140-239, and ICD-10^32^ codes C00-D48. Individual malignancies were categorised as follows (ordered by ICD-9 code): oesophagus (ICD-9 code 150, ICD-10 code C15), stomach (ICD-9 code 151, ICD-10 code C16), colorectal (ICD-9 codes 153-154, ICD-10 codes C18-C20), liver (ICD-9 code 155, ICD-10 code C22), pancreas (ICD-9 code 157, ICD-10 code C25), lung (ICD-9 codes 162, ICD-10 codes C34), mesothelioma (ICD-9 codes 163, ICD-10 code C45), breast (female) (ICD-9 code 174, ICD-10 code C50), ovary (women) (ICD-9 code 183, ICD-10 code C56), prostate (men) (ICD-9 code 185, ICD-10 code C61), bladder (ICD-9 code 188, ICD-10 code C67), kidney (ICD-9 code 189, ICD-10 codes C64 and C65), central nervous system (ICD-9 code 191 and 192, ICD-10 codes C70-C72), non-Hodgkin’s lymphoma (ICD-9 codes 200 and 202, ICD-10 codes C82-C86), multiple myeloma (ICD-9 code 203, ICD-10 code C90.0), and leukaemia (ICD-9 codes 204-208, ICD-10 codes C91-C95). A category of cancer related to smoking was based on current evidence.^33^ ^34^

### Patient involvement

No patients were involved in setting the present research question nor the outcome measures, nor were they involved in developing plans for recruitment, design, or implementation of the study. No patients were asked to advise on interpretation or writing up of results. There are no plans to disseminate the results of the research to study participants or the relevant patient community.

### Statistical analyses

We used raw data for all study years, with the exception of 1996 and 2007, when a psychological distress scale was not administered. In preliminary analyses, we were able to determine that the proportional hazards assumption had not been violated by inspecting the survival curves according to distress categories. With there also being no evidence of an interaction by sex for the association between psychological distress and cancer (P=0.63), we pooled data for men and women and adjusted the effect estimates for sex. Cox proportional hazards models^35^ were used to compute study specific hazard ratios with accompanying 95% confidence intervals for the association between distress and each cancer mortality outcome. We used calendar time (months) as the time scale, with survivors having a censoring date of 15 February 2011. Hazard ratios were minimally adjusted (age and sex only) and maximally adjusted (age, sex, BMI, educational attainment, smoking status, and frequency of alcohol consumption). In the main analyses, we omitted cancer endpoints with too few cases (<50) to provide stable effect estimates.

Subgroup analyses were conducted with data that were available only in selected surveys. Here, age and sex adjusted hazard ratios for the association between distress and cancer were additionally adjusted for physical activity,^36^ C reactive protein,^37^ area level deprivation,^38^ and harmful levels of alcohol intake^39^ in turn, all of which have been linked with selected malignancies featured in the present analyses. We also constructed models using cancer incidence (for SHS only) to compare with results for distress and mortality in this group of studies.

We used the I^2^ statistic as a measure of the degree of inconsistency of effect estimate (heterogeneity) across studies (and cancer outcomes). Although preliminary analyses showed that the I^2^ statistic between studies varied between 0% and 38% depending on the cancer mortality outcome under investigation, we pooled the study specific effect estimates and their standard errors in random effects meta-analyses to provide conservative effect estimates. All analyses were computed with *R* version 3.2.2, with the exception of data imputation, which was performed with SPSS (version 22). 

## Results

Tables 1 and 2[Table tbl1 tbl2] show the characteristics of study members according to each of the 16 included studies. Individual study sample size ranged from 7405 to 14 573 people; there was no difference in mean psychological distress score across the studies.

**Table 1 tbl1:** Characteristics of participants according to individual cohort studies: 13 cohort studies from health survey for England

	**1994**	**1995**	**1997**	**1998**	**1999**	**2000**	**2001**	**2002**	**2003**	**2004**	**2005**	**2006**	**2008**	**No of participants**
No of adults irrespective of consent status	15 804	16 055	8582	15 908	13 947	11 025	15 647	10 331	14 836	12 758	10 303	14 142	15102	174 440
Household response (%)	77	78	76	74	76	75	74	74	73	72	74	68	64	—
Consented to mortality linkage (%)	95.6	93.7	93.9	94.6	90.1	71.9	88.4	88.9	87.3	75.7	80.6	82.6	78.2	—
Included in sample for analysis	14 522	14 573	7654	14 157	11 441	7405	13 107	8717	12 217	8750	7649	11 277	11409	142 878
Mean (SD) follow-up duration (years)	15.1 (3.8)	14.3 (3.4)	12.7 (2.7)	11.8 (2.5)	11.2 (1.8)	9.4 (2.7)	9.2 (1.5	8.4 (1.1)	7.4 (1.0)	6.4 (0.7)	5.4 (0.8)	4.5 (0.5)	2.5 (0.3)	142 878
Deaths from cancer	687	727	297	529	244	258	336	144	211	75	129	85	40	142 878
Mean (SD) age at baseline (years)	45.6 (18.5)	46.0 (18.4)	45.8 (18.0)	46.3 (18.3)	43.5 (17.7)	50.9 (20.7)	46.9 (18.0)	38.9 (19.3)	47.2 (17.9)	44.9 (17.5)	53.9 (19.5)	48.8 (18.0)	48.4 (18.2)	142 878
Women (%)	54.1	54.1	53.8	54.6	53.8	55.7	54.5	55.7	55.2	56.0	54.9	54.9	55.2	142 878
Mean (SD) distress score	1.5 (2.6)	1.6 (2.7)	1.5 (2.6)	1.5 (2.6)	1.7 (2.7)	1.5 (2.7)	1.3 (2.4)	1.6 (2.6)	1.3 (2.5)	1.4 (2.6)	1.2 (2.4)	1.3 (2.5)	1.3 (2.6)	142 878
Distress score ≥7 (%)	6.9	7.8	7.1	7.3	8.3	7.5	5.8	7.4	6.0	6.7	5.3	6.2	6.5	142 878
Left school after minimum leaving age (%)	62.4	61.9	64.1	64.9	70.5	63.6	68.8	77.8	71.0	76.2	64.5	73.1	74.1	142 790
Current smoker (%)	27.4	27.6	28.3	28.0	25.6	24.9	25.5	27.8	24.8	21.7	21.1	22.1	21.8	142444
Mean (SD) BMI	25.9 (4.5)	26.0 (4.5)	26.3 (4.7)	26.4 (4.7)	26.1 (4.8)	26.6 (4.8)	26.9 (4.9)	26.0 (5.0)	26.9 (5.0)	26.7 (5.0)	27.2 (4.9)	27.2 (5.1)	27.2 (5.1)	129 443
Drinks alcohol at least weekly	62.6	64.6	66.0	65.9	52.2	61.6	65.7	64.9	65.4	50.5	62.9	61.4	60.6	126 022

**Table 2 tbl2:** Characteristics of participants according to individual cohort studies: three cohort studies from Scottish health survey

	**1995**	**1998**	**2003**	**No of participants**
No of adults irrespective of consent status	7932	9040	8092	25 064
Household response	—	77	67	—
Consented to mortality linkage	85.3	86.9	87.9	—
Included in analytic sample	6484	7532	6469	20 485
Mean (SD) follow-up duration (years)	13.8 (2.0)	10.7 (1.8)	5.7 (0.8)	20 485
Deaths from cancer	194	281	116	20 485
Cancer incidence	484	590	292	20 485
Mean (SD) age at baseline (years)	40.5 (13.2)	45.3 (15.6)	49.0 (17.3)	20 485
Women (%)	55.0	55.9	56.1	20 485
Mean (SD) distress score	1.7 (2.8)	1.6 (2.8)	1.4 (2.8)	20 485
Distress score ≥7	8.5	8.5	7.9	20 485
Left school after minimum leaving age (%)	66.0	62.6	65.7	20 468
Current smoker (%)	37.3	34.9	26.7	20 356
Mean (SD) BMI	26.1 (4.6)	26.7 (4.9)	27.3 (5.1)	18 379
Drinks alcohol at least weekly	63.0	61.0	60.7	18 227

Figure 1 shows the flow of participants from study induction through to analytical sample. About 18% (n=36 141) of study members were excluded between recruitment and analyses, largely because of refusal to be linked to death or cancer registration records. Individuals with an extant cancer diagnosis at baseline (n=3875) were also excluded. This resulted in a maximum analytic sample of 163 363 (55% women, mean age 46.3 (range 16-102)).

**Figure f1:**
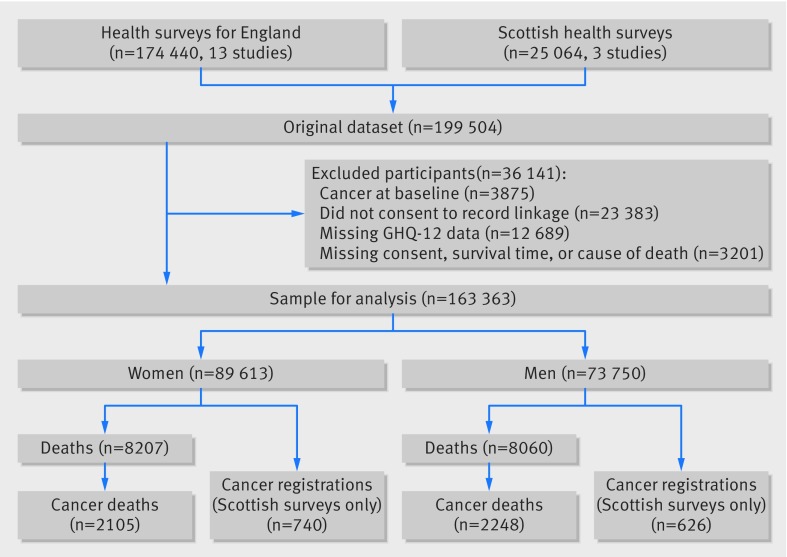
**Fig 1** Study members from induction through to sample for analysis: follow-up of 16 cohort studies from health survey for England and Scottish health survey (n=163 363). People excluded can fall into more than one category so total exceeds 36 141

Table 3 compares the characteristics of the analytical sample with study members who had been excluded. In general, absolute differences were small, though significance at conventional levels was common because of the high numbers of people in the analyses. On this basis, there seemed to be little evidence of selection bias. In the analytical sample, participants were around middle age at study induction (mean age 46.3; range 16-102); around half were women (54.9%), and about a quarter (26.3%) were smokers. Around two thirds of the sample left school after the mandatory age.

**Table 3 tbl3:** Baseline characteristics of survey participants included and excluded from analyses: 16 cohort studies from health survey for England and Scottish health survey

	Included	Excluded	P value
No of study members	163 363	36 141	—
Mean (SD) distress score	1.5 (2.6)	1.5 (2.7)	0.12
Mean (SD) age (years)	46.3 (18.3)	51.9 (21.4)	<0.001
Women (%)	54.9	58.2	<0.001
Mean (SD) BMI	26.6 (4.8)	26.5 (4.9)	0.009
Left school after minimum leaving age (%)	67.9	61.1	<0.001
Current smoker (%)	26.3	26.1	0.09
Drinks alcohol at least weekly (%)	62.0	49.1	<0.001

Based on the 163 363 study members in the sample for analysis, we examined baseline covariates according to the four categories of psychological distress (table 4). As anticipated, study members with higher distress scores had less favourable levels of a range of characteristics, some of which are known risk factors for selected cancers. Thus, relative to people with lower distress levels, the more distressed study members were more likely to have a basic education, smoke, and be obese. The only exception to this observation was the weekly intake of alcohol beverages, which was lower in people reporting higher levels of distress.

**Table 4 tbl4:** Baseline psychological distress score according to other baseline characteristics of study members: health survey for England and Scottish health survey (n=163 363)

	Distress score^*^				P value
	0	1-3	4-6	7-12	
No of people	97 273	41 466	13 114	11 510	—
Mean (SD) age (years)	47.0 (18.0)	45.3 (19.0)	44.9 (18.8)	46.6 (17.4)	<0.001
Female (%)	52.0	56.8	62.4	63.5	<0.001
Obese (%)	20.4	20.5	21.1	23.7	<0.001
Left school after minimum leaving age (%)	68.7	68.2	66.7	62.1	<0.001
Current smoker (%)	24.0	27.1	31.0	37.2	<0.001
Drinks alcohol at least weekly (%)	63.9	61.4	57.7	53.8	<0.001

*Higher score indicates greater degree of psychological distress

During a mean (SD) follow-up of 9.5 (4.3) years across the 16 studies there were 16 267 deaths, 4353 of which were ascribed to cancer of any site. Figure 2 shows the age and sex adjusted relation between psychological distress and mortality from all cancer sites combined according to each of the 16 studies featured in the present meta-analysis. With the exception of three studies with among the lowest number of cancer deaths (1997, 2005, and 2006 HSE), relative to individuals reporting lower distress scores (0-6), those with higher levels (7-12) experienced increased rates of total cancer mortality, though confidence intervals for all but five studies included unity. An I^2^ statistic of 2% suggests essentially no statistical heterogeneity in the study specific estimates. In the 16 studies in aggregate, after adjustment for age and sex, higher levels of distress were associated with a 32% greater risk of total cancer mortality (hazard ratio 1.32, 95% confidence interval 1.18 to 1.48).

**Figure f2:**
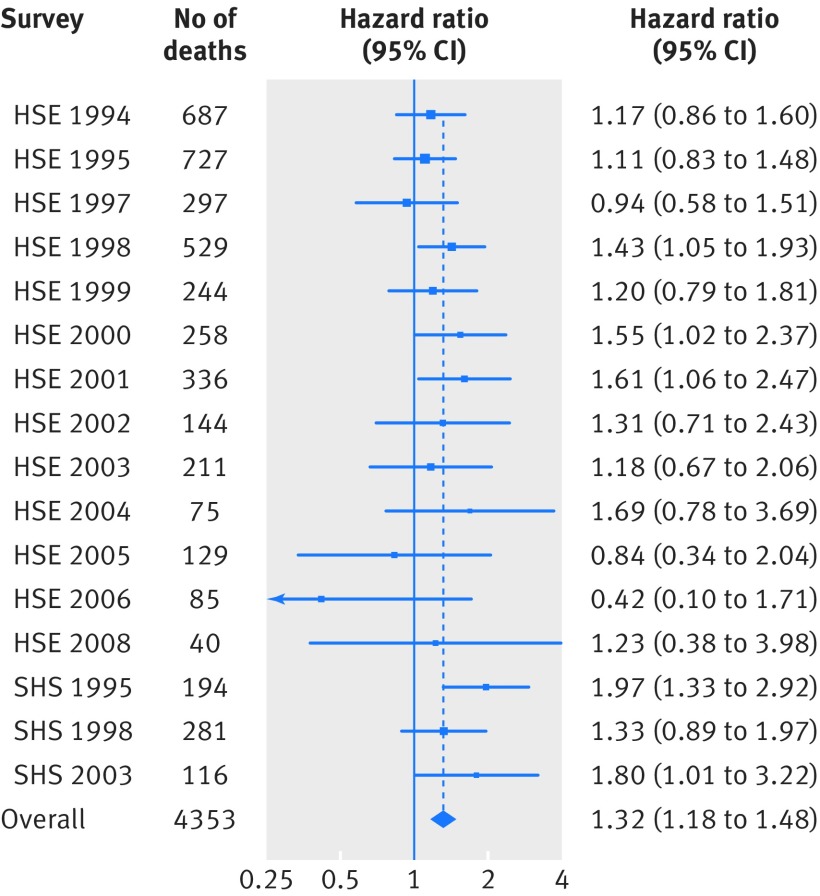
**Fig 2** Hazard ratios (95% confidence intervals) for psychological distress in relation to mortality from all cancers combined according to study: follow-up of 16 cohort studies from health survey for England (HSE) and Scottish health survey (SHS) (n=163 363). Hazard ratios (adjusted for age and sex) are for psychological distress score of 7-12 (most distressed) relative to 0-6. I^2^=2%

Figure 3 shows analyses for distress according to 16 independent (non-overlapping) cancer presentations plus some of these sites in aggregate (total cancer and cancers related and not related to smoking). For all the malignancy endpoints featured in analyses in which hazard ratios were adjusted for age and sex, higher death rates were apparent in people with higher levels of distress, though significance at conventional levels was not always apparent. Thus, of the individual sites, in the model with age and sex, the weakest effects were seen for lung cancer and the strongest for mesothelioma. Some of these estimates were imprecise, as evidenced by the wide confidence intervals because of a low number of cancer deaths. We also show the impact of adjustment for a range of further covariates. Relative to the age and sex adjusted hazard ratios, adjustment for covariates that included socioeconomic position (education) and health behaviours (cigarette smoking, alcohol intake) had little attenuating effect; indeed, in some cases, positive confounding was apparent. One exception was tobacco related cancer (including lung), for which, unsurprisingly, the addition of smoking to the multivariable model led to partial attenuation of the association with distress (table A in appendix 1 shows the impact of control for individual confounding factors in the multivariable model).

**Figure f3:**
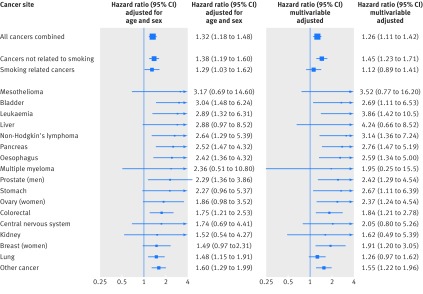
**Fig 3** Hazard ratios (95% confidence interval) for psychological distress in relation to selected cancer death outcomes: follow-up of 16 cohort studies from health survey for England and Scottish health survey (n=163 363). Hazard ratios are for psychological distress score of 7-12 (most distressed) relative to 0-6, and are age and—except in single sex analyses—sex adjusted (I^2^=15%), or multivariable adjusted (age, sex, BMI, educational attainment, smoking status, and alcohol consumption; I^2^=37%)

Table 5 shows the four categories of psychological distress we used to explore dose-response associations with different presentations of cancer. In these analyses there was some evidence of stepwise effects across the full distress range for cancer of the colorectum and prostate. With different models being based on different analytical samples because of missing covariates, we recomputed these effects estimates in a non-missing dataset—that is, the same sample size in both models—and our results were unchanged.

**Table 5 tbl5:** Hazard ratios (95% confidence interval) for association between psychological distress and mortality from cancer: follow-up of 16 cohort studies from health survey for England and Scottish health survey (n=163 363)

Cancer and adjustment	Deaths^*^	No of people	Distress score				P value for trend	Distress score (7-12 *v* 0-6)
			0	1-3	4-6	7-12		
**All cancers**								
Age and sex	4353	163 363	1 (ref)	0.99 (0.92 to 1.08)	1.05 (0.91 to 1.20)	1.31 (1.16 to 1.49)	<0.001	1.32 (1.18 to 1.48)
Multivariable^†^	3800	146 008	1	0.95 (0.88 to 1.03)	0.93 (0.78 to 1.10)	1.22 (1.07 to 1.38)	0.04	1.26 (1.11 to 1.42)
**Not related to smoking**								
Age and sex	2323	163 363	1	0.96 (0.87 to 1.06)	1.06 (0.89 to 1.27)	1.36 (1.17 to 1.59)	<0.001	1.38 (1.19 to 1.60)
Multivariable	2036	146 008	1	0.94 (0.84 to 1.04)	1.01 (0.82 to 1.25)	1.43 (1.21 to 1.69)	<0.001	1.45 (1.23 to 1.71)
**Smoking related**								
Age and sex	2030	163 363	1	1.03 (0.93 to 1.15)	1.09 (0.86 to 1.38)	1.29 (1.02 to 1.64)	0.03	1.29 (1.03 to 1.62)
Multivariable	1764	146 008	1	0.98 (0.88 to 1.09)	0.93 (0.76 to 1.14)	1.09 (0.86 to 1.38)	0.92	1.12 (0.89 to 1.41)
**Lung**								
Age and sex	992	163 363	1	1.10 (0.94 to 1.28)	1.45 (1.05 to 2.00)	1.55 (1.17 to 2.06)	<0.001	1.48 (1.15 to 1.91)
Multivariable	865	146 008	1	1.04 (0.88 to 1.22)	1.21 (0.90 to 1.64)	1.26 (0.95 to 1.66)	0.13	1.26 (0.97 to 1.62)
**Colorectal**								
Age and sex	391	163 363	1	1.09 (0.86 to 1.39)	1.48 (1.02 to 2.14)	1.77 (1.20 to 2.59)	0.01	1.75 (1.21 to 2.53)
Multivariable	341	135 888	1	1.11 (0.86 to 1.44)	1.56 (1.05 to 2.33)	1.89 (1.23 to 2.90)	0.01	1.84 (1.21 to 2.78)
**Breast (female)**								
Age	257	89 613	1	0.93 (0.63 to 1.37)	0.74 (0.38 to 1.44)	1.36 (0.87 to 2.13)	0.97	1.49 (0.97 to 2.31)
Multivariable	213	78 707	1	1.03 (0.66 to 1.60)	0.97 (0.47 to 1.99)	1.69 (1.04 to 2.73)	0.41	1.91 (1.20 to 3.05)
**Prostate**								
Age	253	73 750	1	0.87 (0.62 to 1.22)	1.91 (1.21 to 3.01)	2.28 (1.33 to 3.92)	0.004	2.29 (1.36 to 3.86)
Multivariable	216	67 301	1	0.93 (0.64 to 1.35)	1.81 (0.93 to 3.52)	2.38 (1.25 to 4.57)	0.01	2.42 (1.29 to 4.54)
**Pancreas**								
Age and sex	244	163 363	1	1.18 (0.87 to 1.59)	1.32 (0.75 to 2.33)	2.09 (1.19 to 3.65)	0.35	2.52 (1.47 to 4.32)
Multivariable	218	146 008	1	1.16 (0.84 to 1.59)	1.12 (0.55 to 2.26)	2.08 (1.11 to 3.89)	0.772	2.76 (1.47 to 5.19)
**Oesophagus**								
Age and sex	209	163 363	1	1.09 (0.79 to 1.52)	1.13 (0.51 to 2.50)	2.43 (1.33 to 4.44)	0.314	2.42 (1.36 to 4.32)
Multivariable	174	135 888	1	1.02 (0.71 to 1.47)	1.34 (0.60 to 3.00)	2.48 (1.25 to 4.92)	0.38	2.59 (1.34 to 5.00)
**Stomach**								
Age and sex	156	155 714	1	0.89 (0.60 to 1.32)	0.83 (0.33 to 2.10)	2.22 (0.91 to 5.39)	0.72	2.27 (0.96 to 5.37)
Multivariable	141	139 323	1	0.90 (0.60 to 1.37)	0.84 (0.30 to 2.37)	2.52 (1.03 to 6.20)	0.97	2.67 (1.11 to 6.39)
**Ovary**								
Age	125	83 424	1	0.93 (0.59 to 1.47)	1.53 (0.76 to 3.07)	1.71 (0.88 to 3.32)	0.14	1.86 (0.98 to 3.52)
Multivariable	111	67 823	1	0.96 (0.60 to 1.55)	1.87 (0.81 to 4.27)	2.18 (1.11 to 4.28)	0.04	2.37 (1.24 to 4.54)
**Bladder**								
Age and sex	121	163 363	1	1.44 (0.93 to 2.22)	2.42 (1.10 to 5.31)	3.31 (1.55 to 7.09)	0.01	3.04 (1.48 to 6.24)
Multivariable	107	146 008	1	1.27 (0.79 to 2.05)	1.92 (0.81 to 4.57)	2.55 (1.02 to 6.35)	0.18	2.69 (1.11 to 6.53)
**Non-Hodgkin’s lymphoma**								
Age and sex	110	151 954	1	0.77 (0.44 to 1.37)	1.19 (0.46 to 3.10)	1.99 (0.97 to 4.10)	0.46	2.64 (1.29 to 5.39)
Multivariable	99	135 888	1	0.70 (0.38 to 1.27)	1.22 (0.46 to 3.24)	2.46 (1.06 to 5.73)	0.33	3.14 (1.36 to 7.24)
**Central nervous system**								
Age and sex	105	154 613	1	1.10 (0.67 to 1.81)	1.80 (0.83 to 3.91)	2.05 (0.78 to 5.41)	0.10	1.74 (0.69 to 4.41)
Multivariable	96	138 348	1	1.07 (0.63 to 1.83)	2.02 (0.92 to 4.42)	2.46 (0.91 to 6.63)	0.06	2.05 (0.80 to 5.26)
**Leukaemia**								
Age and sex	101	163 363	1	0.99 (0.60 to 1.66)	3.08 (1.16 to 8.15)	2.61 (1.06 to 6.44)	0.11	2.89 (1.32 to 6.31)
Multivariable	87	135 999	1	1.13 (0.65 to 1.97)	3.86 (0.72 to 20.8)	3.92 (1.36 to 11.2)	0.06	3.86 (1.42 to 10.5)
**Kidney**								
Age and sex	90	163 363	1	0.72 (0.39 to 1.34)	1.40 (0.54 to 3.66)	1.22 (0.43 to 3.45)	0.82	1.52 (0.54 to 4.27)
Multivariable	79	127 961	1	0.80 (0.43 to 1.51)	1.67 (0.63 to 4.40)	1.40 (0.41 to 4.77)	0.79	1.62 (0.49 to 5.39)
**Liver**								
Age and sex	61	146 997	1	2.24 (1.12 to 4.48)	3.67 (1.12 to 12.0)	3.09 (0.97 to 9.90)	0.07	2.88 (0.97 to 8.52)
Multivariable	53	125 978	1	1.57 (0.86 to 2.85)	NA	1.73 (0.53 to 16.3)	0.20	4.24 (0.66 to 27.0)
**Multiple myeloma**								
Age and sex	51	135 555	1	0.60 (0.27 to 1.34)	2.07 (0.42 to 10.2)	1.80 (0.37 to 8.7)	0.72	2.36 (0.51 to 10.8)
Multivariable	41	111 534	1	0.70 (0.25 to 1.99)	4.79 (0.42 to 55.3)	1.41 (0.18 to 11.3)	0.83	1.95 (0.25 to 15.5)
**Mesothelioma**								
Age and sex	51	134 208	1	1.09 (0.49 to 2.41)	2.38 (0.27 to 20.7)	3.42 (0.68 to 17.1)	0.80	3.17 (0.69 to 14.6)
Multivariable	51	120 461	1	1.12 (0.51 to 2.50)	2.39 (0.27 to 21.6)	3.91 (0.78 to 19.6)	0.62	3.52 (0.77 to 16.2)
**“Other” cancers**								
Age and sex	1036	163 363	1	1.08 (0.93 to 1.25)	1.36 (1.09 to 1.70)	1.66 (1.33 to 2.08)	<0.001	1.60 (1.29 to 1.99)
Multivariable	908	146 008	1	1.01 (0.86 to 1.19)	1.30 (1.01 to 1.67)	1.58 (1.24 to 2.01)	<0.001	1.55 (1.22 to 1.96)

NA=too few cancer deaths to run analyses.

*No of deaths and total numbers in each model varies for sex specific cancers and when no participants died with particular cancers in individual survey years resulting in that whole cohort study being excluded from meta-analysis.

†Hazard ratio adjusted for age, sex (except in single sex analyses), BMI, educational attainment, smoking status, and frequency of alcohol consumption. For selected outcomes sample for analysis is markedly smaller than for all cancers combined because certain cohort studies, particularly those with shorter duration of follow-up, had not accumulated any deaths for endpoint of interest.

We then carried out some planned subgroup analyses. Firstly, as described, certain potential covariates (physical activity, C reactive protein, area based deprivation, quantity of alcohol consumed) were collected only in selected studies and therefore did not feature in our main analyses. Because of the smaller numbers, we were able to examine the impact of adjustment for these covariates only on the relation between distress and all cancers combined (table B in appendix 1). The strength of the relation between distress and total cancer was little changed. Secondly, to explore the role of reverse causality—people entering the studies might have some symptoms of undiagnosed cancer, including pain and tiredness, which could cause, or be taken for, mental distress—we excluded study members who died in the first five years of follow-up from the particular endpoint featured in each analysis. In doing so, we found that most of the associations between distress and cancer were largely unaffected (fig A in appendix 2).

Thirdly, in related analyses, given that death data combine both incidence and survival, we examined if there was a relation between distress and incidence based on registration of a cancer diagnosis (data available only for the three Scottish studies) as this is more proximal to the exposure of interest and therefore the analyses potentially provide greater insights into aetiology. Figure 4 shows that there was some evidence of differential effects for cancer of all locations combined and colorectal cancer, such that the associations between distress and incidence were weaker, though the latter analysis particularly was compromised by relatively few events.

**Figure f4:**
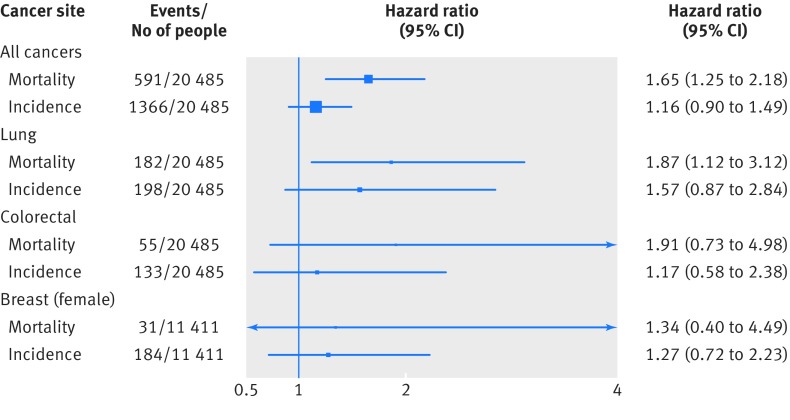
**Fig 4** Hazard ratios (95% confidence interval) for psychological distress in relation to selected cancer outcomes: comparison of effects for incidence and mortality in follow-up of three cohort studies from Scottish health survey (SHS; n=20 485). Hazard ratios (adjusted for age and sex) are for psychological distress score of 7-12 (most distressed) relative to 0-6. Individuals with cancers registered before baseline (n=696) were excluded from analyses of cancer incidence

Lastly, 18% (n=36 141) of study members were excluded between recruitment and analyses, largely because of refusal to be linked to death or cancer registration records (fig 1). Accordingly, for each of the 16 cohort studies in the analysis we used multiple multivariate imputation based on baseline variables available for any missing values. We ran five cycles of regression, which generated five imputation datasets for each of the 16 studies, and the results were obtained by averaging results across each of these five datasets using the approach of Rubin.^40^ This procedure takes into account the uncertainty in the imputation process as well as uncertainty from random variation. Meta-analysis of the results from these imputed study specific data (table C in appendix 1) gave similar results to those seen with the non-missing dataset as reported in the present paper. 

## Discussion

### Principal findings

In this pooling of unpublished individual participant data, we found that people in the highest distress grouping relative to the lowest experienced increased rates of death from selected cancers. Thus, after adjustment for covariates that are known risk factors for selected malignancies, such as adverse health behaviours, and with reverse causality (by left censoring) and missing data (by imputation) taken into consideration, the most consistently robust effects were evident for carcinoma of the colorectum, prostate, pancreas, and oesophagus and for leukaemia. For two of these malignancies—colorectal and prostate—a gradient was apparent, such that the greater level of distress, the higher the risk of cancer mortality. These associations provide partial support for our hypotheses based on plausible mechanisms of effect.

### Usefulness of the present study

Our findings could be important in advancing understanding of the role of psychological distress in cancer aetiology and cancer progression as investigators attempt to ascertain what role this and other psychological factors (such as psychosocial stress, cognition, personality, life satisfaction) have, if any, in prevention and prognosis. By contextualising the predictive value of distress for risk of cancer by comparing it with established non-psychological risk factors using data from the present study (table D in appendix 1), it is evident that, aside from lung cancer for which cigarette smoking has a known causal influence, the hazard ratios for higher levels of distress are of similar magnitude to those for current smoking and obesity for selected cancer presentations. Individually, however, none of these risk factors is powerful enough to determine a person’s risk: in analyses of death from a common cancer such as colorectal for instance, the sensitivity—the proportion of people who went on to develop a disease who also had the risk factor at baseline—was only 8% for psychological distress, 17% for current cigarette smoking, and 27% for obesity. As has been shown for cardiovascular disease, however, where multifactorial algorithms are in widespread use in general practice (such as Framingham,^41^ QRisk^42^), collectively, these and other risk factors might have predictive utility for common cancer presentations (such as colorectal, breast, prostate). In developing such algorithms, psychological distress could be considered as a component, which is not currently the case.^43^ That these risk factors collectively have predictive value for selected cancers, together with the well established observations that cancers rates differ systematically across time,^44^ location,^45^ and migration pattern,^46^ strongly suggest that the initiation of cancers is not a simple stochastic process reflecting the number of tissue specific stem cell divisions,^47^ as has been suggested.^48^

### Study strengths and limitations

Our study has some strengths, including the use of unpublished raw data from similarly conducted studies in the general population; as such, our findings are not subject to publication bias and comparison across studies is straightforward. We also used a large and well characterised dataset relative to many other studies in this specialty. Our work is of course not without its limitations. The assessment of psychological distress with the GHQ-12 referenced the preceding four week period. A short bout of distress is unlikely to be of aetiological relevance for a disease like cancer, which has a long induction period. There is evidence, however, that rates of recurrence are high for psychological distress. For instance, in a population of 4363 people in a similar age range to the present study members, we found that, based on the general health questionnaire (30 items) over a maximum of 19 years of surveillance (four phases of data collection), two thirds of the sample classified as distressed at baseline were also distressed on one or more occasion during follow-up. This is broadly consistent with findings for clinical depression.^49^ Thus, a single administration of a distress inventory seems to capture cases of long term depression and anxiety. This notwithstanding, having serial measurement of our exposure would have provided further insights into the chronicity of psychological distress and would have the added advantage of allowing us to mimic a trial in an observational context by identifying a group whose depression resolved over time and observing the occurrence of cancer in this group. 

While we chose to include an array of cancer outcomes to explore specificity of association, not all of these were hypothesis driven. It is also the case that, given that our meta-analysis is based on observational studies, as well characterised as these studies were, confounding by known or unknown factors remains a perennial concern. The assessment of dietary characteristics, for example, was an omission. This problem could theoretically be circumvented in a randomised controlled trial of people undergoing treatment for depression and anxiety who are also subject to surveillance for cancer events where, if it is genuinely causal, a reversal of symptoms of distress would produce a lower rate of cancer in treated patients. While such an aetiological trial has been conducted in the context of cardiovascular disease— reduction in depression produced a lower risk of total mortality in one^50^ but had no impact on myocardial reinfarction rates in another^51^—the logistics involved with the size and duration of a trial for multiple cancer presentations are likely to be prohibitive. An alternative but related approach would be to use Mendelian randomisation in the context of observational data in which a gene variant for psychological distress in principle provides an unconfounded estimate of the relation between an exposure and a disease outcome.^52^ As the genes for depression get identified,^53^ this represents a realistic proposition; unfortunately, the studies that comprise the present collaboration did not capture genetic material.

### Comparison with other studies

The present analyses considerably extend our existing work,^21^ where we have shown that higher levels of distress were related to major causes of mortality, including cardiovascular disease, external causes of death, and all cancers combined, by exploring the link between distress and 16 different cancer presentations. We are not aware of previous meta-analyses on symptoms of psychological distress in relation to site specific cancer. In a recent meta-analysis of the occurrence of cancer subsequent to the assessment of major depression,^13^ which used study rather than individual level data and excluded studies in which investigators captured depressive symptoms, the aggregated result for depression and cancer for all malignancies across the 13 studies included unity (relative risk 1.12, 95% confidence interval 0.99 to 1.26). This was of markedly lower magnitude than our estimate between psychological distress and overall cancer (multivariable adjusted hazard ratio 1.32, 95% confidence interval 1.18 to 1.48). The discordant findings of prospective studies published since that meta-analysis have not clarified matters.^54^ ^55^ ^56^ ^57^ ^58^ ^59^ ^60^ Even within the same study, all cancers combined showed opposing gradients with depression in sex stratified analyses.^59^ Studies of sufficient scale to explore site specific associations with depression are rare, and the few that have been conducted show null effects for colon, lung, and prostate.^57^ ^60^

### Mechanisms of effect

Cancer mortality according to anatomical site—the main outcome in our study—is a composite of the onset of cancer (aetiology) together with survival from the disease (prognosis). The influence of psychological distress on processes acting at either or both of these disease stages could therefore influence the risk of cancer mortality. Moreover, these mechanisms can be direct (biological) and/or indirect (behavioural), and their action cancer specific and/or common to multiple presentations.

People with chronic distress typically have a less favourable lifestyle relative to those with lower levels,^12^ and this has been advanced as one means by which distress can be embodied, so increasing the risk of cancer. While we controlled for cigarette smoking, heavy alcohol intake, and physical inactivity in our analyses—and the associations held—health seeking behaviours might also be important, perhaps at a later stage in the disease process. Thus, people who are distressed might be less likely to comply with requests for screening,^61^ resulting in a delayed diagnosis, and, once cancer is diagnosed, depression might hamper adherence to treatment.^62^ These findings, however, are notuniversal.^63^ We did not collect data on treatment behaviours in the present study.

Of the biological mechanisms, mood disorders such as depression have been implicated in immune pathways and are known to provoke inflammatory responses. Prolonged immune dysregulation can compromise the repair capacity of the exposed cells, potentially contributing to genetic instability and mutations, alterations in DNA repair, and inhibition of apoptosis.^64^ ^65^ Immune dysregulation can also lead to a worse prognosis for several carcinomas, including cancer of the colorectum, lung, mesothelium, and stomach.^66^ Depression and distress are also associated with markers of increased inflammation, such as interleukin 6, high sensitivity C reactive protein, and soluble tumour necrosis factor receptor.^67^ In subgroup analyses, the associations between distress and cancer we observed were unchanged after the addition of circulating C reactive protein concentrations to the multivariable model, but we did not have data on a wider suite of inflammatory indicators. The lack of specificity of the relation between distress and cancer site in our analyses did not provide unambiguous insights into potential mechanisms, though, in general, the associations seemed stronger for some hormone related cancers, such as carcinoma of the prostate and ovaries. This observation accords with the notion of stress related mechanisms, which include dysregulation of the hypothalamic pituitary adrenal (HPA)^68^ and sympathetic adrenal medullary (SAM) axes.^69^

### Exploring the role of reverse causality

It is plausible that the associations we found between distress and cancer reflect both the effects of cancer—diagnosed and undiagnosed—on mood, the effects of distress on cancer progression, or a combination. As described, it is well documented that a diagnosis of cancer can give rise to distress,^70^ and we dealt with this potential source of reverse causality by using the standard practice of excluding members with self reported malignancy at study entry. Having done so, when we explored the risk between distress and total cancer according to study, those with longer follow-up generally showed weaker associations. As duration of follow-up increases, the proportion of surviving people who had entered the study with unknown cancer diminishes relative to the total number of deaths from cancer; the influence of cancer on distress should likewise wane over time. Moreover, in analyses of the Scottish studies, the associations were somewhat weaker for cancer incidence than for cancer mortality, though these analyses were not well powered because of the lower number of new cancer cases. Taking these observations together, there was a suggestion that subclinical malignancy might have had an impact on mood. Thus, to explore the impact of occult cancer, we excluded study members who died in the first five years of follow-up. This practice is based on the assumption that people with occult cancers of the more lethal variety will have died during this period. In these analyses, the gradients between distress and cancer were, however, still seen.

In conclusion, our findings add to the growing evidence of an association between psychological distress and physical conditions by characterising new relations with death from selected cancer presentations. The extent to which these associations could be causal requires further testing with alternative study designs.

**What is already known on this topic**

While psychological distress (symptoms of depression and anxiety) is related to increased rates of cardiovascular disease, links with different presentations of cancer are unclear and, for selected malignancies, untested

**What this study adds**

A pooled analysis of unpublished raw data from 16 prospective cohort studies suggests associations between distress and cancer, most notably for carcinoma of the colorectum, prostate, pancreas, and oesophagus and for leukaemia
This adds to the growing evidence that psychological distress could have some predictive capacity for certain somatic diseases
With extant evidence being exclusively based on observational studies, further research is now required to clarify the extent to which each of the associations between distress and cancer is likely to be causal.

## Notes

We thank Robert Miller for his advice and correction.

Ethical approval: The study was approved by the London research ethics council.

Data sharing: The baseline data for the surveys described herein are curated by the UK Data Archive at the University of Essex, and can be downloaded free of charge for non-commercial purposes from the Economic and Social Data Service (www.esds.ac.uk/findingData/hseTitles.asp). Syntax for the present analyses are available from the authors.

Transparency: The lead author affirms that the manuscript is an honest, accurate, and transparent account of the study being reported; that no important aspects of the study have been omitted; and that any discrepancies from the study as planned have been explained.
